# Chlorido(2-methyl-4-oxo-4*H*-pyran-3-olato-*κ* ^2^ *O* ^3^,*O* ^4^)(1,10-phenanthroline-*κ* ^2^ *N*,*N*′)copper(II)

**DOI:** 10.1107/S1600536808023829

**Published:** 2008-07-31

**Authors:** Kong Wai Tan, Chew Hee Ng, Mohd Jamil Maah, Seik Weng Ng

**Affiliations:** a Department of Chemistry, University of Malaya, 50603 Kuala Lumpur, Malaysia; b Faculty of Engineering and Science, Universiti Tunku Abdul Rahman, 53300 Kuala Lumpur, Malaysia; c Department of Chemistry, University of Malaya, 50603 Kuala Lumpur, Malaysia

## Abstract

The copper(II) atoms in the two independent mol­ecules of the title compound, [Cu(C_6_H_5_O_3_)Cl(C_12_H_8_N_2_)], both adopt square-pyramidal geometries. The two coordinating atoms of the two heterocyclic ligands comprise the square plane, and the chlorine atom occupies the apical position of the coordination environment.

## Related literature

For the structure of aqua­chlorido(maltolato)copper hydrate, which adopts a chlorido-bridged chain structure, see: Odoko *et al.* (2002 ▶).
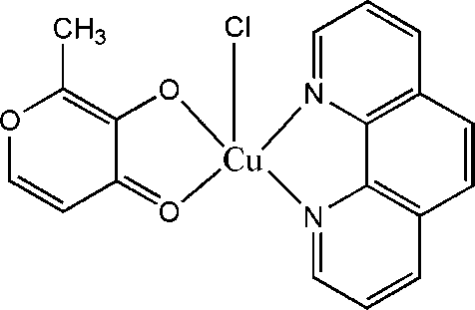


## Experimental

### 

#### Crystal data

[Cu(C_6_H_5_O_3_)Cl(C_12_H_8_N_2_)]
*M* *_r_* = 404.29
Triclinic, 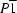

*a* = 9.0043 (2) Å
*b* = 12.0599 (2) Å
*c* = 14.4655 (3) Å
α = 77.412 (1)°
β = 87.380 (1)°
γ = 84.565 (1)°
*V* = 1525.66 (5) Å^3^
*Z* = 4
Mo *K*α radiation
μ = 1.63 mm^−1^
*T* = 100 (2) K
0.15 × 0.05 × 0.05 mm

#### Data collection

Bruker SMART APEX diffractometer
Absorption correction: multi-scan (*SADABS*; Sheldrick, 1996 ▶) *T*_min_ = 0.792, *T*_max_ = 0.923
14221 measured reflections
6825 independent reflections
5053 reflections with *I* > 2σ(*I*)
*R*_int_ = 0.041

#### Refinement

*R*[*F* ^2^ > 2σ(*F* ^2^)] = 0.056
*wR*(*F* ^2^) = 0.169
*S* = 1.04
6825 reflections
453 parameters
H-atom parameters constrained
Δρ_max_ = 2.04 e Å^−3^
Δρ_min_ = −1.26 e Å^−3^

### 

Data collection: *APEX2* (Bruker, 2007 ▶); cell refinement: *SAINT* (Bruker, 2007 ▶); data reduction: *SAINT*; program(s) used to solve structure: *SHELXS97* (Sheldrick, 2008 ▶); program(s) used to refine structure: *SHELXL97* (Sheldrick, 2008 ▶); molecular graphics: *X-SEED* (Barbour, 2001 ▶); software used to prepare material for publication: *publCIF* (Westrip, 2008 ▶).

## Supplementary Material

Crystal structure: contains datablocks I, New_Global_Publ_Block. DOI: 10.1107/S1600536808023829/xu2443sup1.cif

Structure factors: contains datablocks I. DOI: 10.1107/S1600536808023829/xu2443Isup2.hkl

Additional supplementary materials:  crystallographic information; 3D view; checkCIF report

## Figures and Tables

**Table 1:** Selected bond lengths (Å)

Cu1—N1	2.001 (3)
Cu1—N2	2.010 (3)
Cu1—O1	1.979 (3)
Cu1—O2	1.920 (3)
Cu1—Cl1	2.540 (1)
Cu2—O4	1.989 (3)
Cu2—O5	1.924 (3)
Cu2—N3	1.999 (3)
Cu2—N4	2.014 (3)
Cu2—Cl2	2.524 (1)

**Fig. 1.**
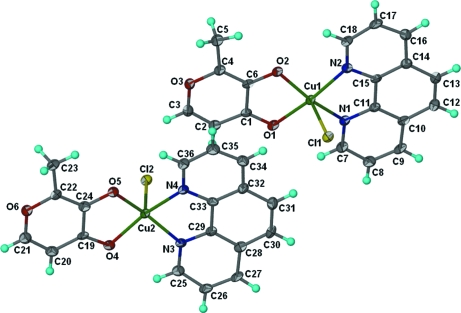
Thermal ellipsoid (Barbour, 2001) plot of the two independent molecules of CuCl(C12H8N2)(C6H5O3) at the 70% probability level. Hydrogen atoms are drawn as spheres of arbitrary radii.

## supplementary crystallographic information

### Comment

There are few crystal structure studies of metal derivatives of maltol. A monochlorocopper(II) derivative is known; the deprotonated ligand chelates to the metal atom; adjacent molecules are linked into a chain by chloride bridges (Odoko *et al.*, 2002). In the present 1,10-phenanthroline adduct (Scheme I), the deprotonated ligand and the *N*-heterocycle both chelate to the copper atom, which shows square pyramidal coordination (Fig. 1 and Table 1). There are two independent molecules in the asymmetric unit.

### Experimental

Solid maltol (0.20 g, 1.6 mmol) was added to a sodium hydroxide solution (0.60 g,1.5 mmol, 20 ml) followed by 1,10-phenanthroline (0.27 g, 1.5 mmol) dissolved in methanol (5 ml). Copper(II) chloride dihydrate ((0.26 g, 1.5 mmol) dissolved in water (10 ml) was added. The green solution was set aside for the growth of crystals.

### Refinement

Carbon-bound H-atoms were placed in calculated positions (C—H 0.95 to 0.98 Å) and were included in the refinement in the riding model approximation, with *U*_iso_(H) set to 1.2 *U*_eq_(C). The final difference Fourier map had a large peak/deep hole at about 1 Å from Cu1.

### Crystal data

**Table:**

[Cu(C_6_H_5_O_3_)Cl(C_12_H_8_N_2_)]	*Z* = 4
*M**_r_* = 404.29	*F*_000_ = 820
Triclinic, *P*1	*D*_x_ = 1.760 Mg m^−^^3^
Hall symbol: -P 1	Mo *K*α radiation λ = 0.71073 Å
*a* = 9.0043 (2) Å	Cell parameters from 4344 reflections
*b* = 12.0599 (2) Å	θ = 2.3–28.2º
*c* = 14.4655 (3) Å	µ = 1.63 mm^−^^1^
α = 77.412 (1)º	*T* = 100 (2) K
β = 87.380 (1)º	Irregular block, green
γ = 84.565 (1)º	0.15 × 0.05 × 0.05 mm
*V* = 1525.66 (5) Å^3^	

### Data collection

**Table:**

Bruker SMART APEX diffractometer	6825 independent reflections
Radiation source: fine-focus sealed tube	5053 reflections with *I* > 2σ(*I*)
Monochromator: graphite	*R*_int_ = 0.041
*T* = 100(2) K	θ_max_ = 27.5º
ω scans	θ_min_ = 1.4º
Absorption correction: Multi-scan(SADABS; Sheldrick, 1996)	*h* = −11→11
*T*_min_ = 0.792, *T*_max_ = 0.923	*k* = −15→15
14221 measured reflections	*l* = −18→18

### Refinement

**Table:**

Refinement on *F*^2^	Secondary atom site location: difference Fourier map
Least-squares matrix: full	Hydrogen site location: inferred from neighbouring sites
*R*[*F*^2^ > 2σ(*F*^2^)] = 0.056	H-atom parameters constrained
*wR*(*F*^2^) = 0.169	*w* = 1/[σ^2^(*F*_o_^2^) + (0.1077*P*)^2^ + 0.3956*P*] where *P* = (*F*_o_^2^ + 2*F*_c_^2^)/3
*S* = 1.04	(Δ/σ)_max_ = 0.001
6825 reflections	Δρ_max_ = 2.04 e Å^−^^3^
453 parameters	Δρ_min_ = −1.26 e Å^−^^3^
Primary atom site location: structure-invariant direct methods	Extinction correction: none

### Fractional atomic coordinates and isotropic or equivalent isotropic displacement parameters (Å^2^)

**Table:**

	*x*	*y*	*z*	*U*_iso_*/*U*_eq_	
Cu1	0.85441 (5)	0.77700 (4)	0.28183 (3)	0.01441 (15)	
Cu2	0.57072 (5)	0.17397 (4)	0.21656 (3)	0.01458 (15)	
O1	0.7016 (3)	0.6822 (2)	0.25382 (19)	0.0172 (6)	
O2	0.8556 (3)	0.6797 (2)	0.40608 (18)	0.0165 (6)	
O3	0.6602 (3)	0.4274 (2)	0.48535 (19)	0.0169 (6)	
O4	0.4569 (3)	0.0598 (2)	0.17636 (19)	0.0169 (6)	
O5	0.6122 (3)	0.0568 (2)	0.32809 (18)	0.0171 (6)	
O6	0.4537 (3)	−0.2134 (2)	0.39503 (19)	0.0180 (6)	
N1	0.8155 (3)	0.9036 (3)	0.1685 (2)	0.0150 (7)	
N2	0.9678 (3)	0.8943 (3)	0.3209 (2)	0.0152 (7)	
N3	0.5841 (4)	0.2733 (3)	0.0869 (2)	0.0160 (7)	
N4	0.7330 (3)	0.2675 (3)	0.2404 (2)	0.0140 (6)	
C1	0.6830 (4)	0.6016 (3)	0.3256 (3)	0.0142 (7)	
C2	0.5859 (4)	0.5143 (3)	0.3298 (3)	0.0168 (8)	
H2A	0.5254	0.5147	0.2776	0.020*	
C3	0.5805 (4)	0.4310 (3)	0.4083 (3)	0.0179 (8)	
H3A	0.5173	0.3720	0.4094	0.021*	
C4	0.7526 (4)	0.5118 (3)	0.4863 (3)	0.0161 (8)	
C5	0.8287 (5)	0.4966 (4)	0.5774 (3)	0.0216 (9)	
H5A	0.7598	0.5234	0.6239	0.032*	
H5B	0.9162	0.5406	0.5679	0.032*	
H5C	0.8603	0.4156	0.6007	0.032*	
C6	0.7679 (4)	0.5981 (3)	0.4088 (3)	0.0147 (8)	
C7	0.7350 (4)	0.9050 (4)	0.0933 (3)	0.0185 (8)	
H7A	0.6753	0.8438	0.0945	0.022*	
C8	0.7352 (5)	0.9933 (4)	0.0127 (3)	0.0198 (8)	
H8A	0.6759	0.9919	−0.0397	0.024*	
C9	0.8216 (4)	1.0823 (4)	0.0096 (3)	0.0181 (8)	
H9A	0.8234	1.1424	−0.0451	0.022*	
C10	0.9075 (4)	1.0833 (3)	0.0883 (3)	0.0158 (8)	
C11	0.8986 (4)	0.9918 (3)	0.1668 (3)	0.0145 (8)	
C12	1.0033 (4)	1.1708 (3)	0.0933 (3)	0.0178 (8)	
H12A	1.0108	1.2333	0.0409	0.021*	
C13	1.0835 (4)	1.1652 (3)	0.1722 (3)	0.0179 (8)	
H13A	1.1480	1.2228	0.1732	0.021*	
C14	1.0723 (4)	1.0742 (3)	0.2534 (3)	0.0165 (8)	
C15	0.9835 (4)	0.9861 (3)	0.2501 (3)	0.0141 (8)	
C16	1.1477 (4)	1.0643 (4)	0.3396 (3)	0.0182 (8)	
H16A	1.2096	1.1212	0.3471	0.022*	
C17	1.1295 (4)	0.9718 (4)	0.4112 (3)	0.0201 (9)	
H17A	1.1781	0.9647	0.4694	0.024*	
C18	1.0403 (4)	0.8874 (3)	0.4002 (3)	0.0167 (8)	
H18A	1.0312	0.8231	0.4508	0.020*	
C19	0.4546 (4)	−0.0287 (3)	0.2423 (3)	0.0143 (8)	
C20	0.3761 (4)	−0.1249 (4)	0.2403 (3)	0.0185 (8)	
H20A	0.3229	−0.1279	0.1858	0.022*	
C21	0.3780 (4)	−0.2119 (4)	0.3169 (3)	0.0191 (8)	
H21A	0.3233	−0.2750	0.3154	0.023*	
C22	0.5334 (4)	−0.1241 (3)	0.4005 (3)	0.0170 (8)	
C23	0.6088 (5)	−0.1400 (4)	0.4921 (3)	0.0203 (9)	
H23A	0.6762	−0.2100	0.5018	0.030*	
H23B	0.5338	−0.1457	0.5438	0.030*	
H23C	0.6662	−0.0747	0.4913	0.030*	
C24	0.5369 (4)	−0.0313 (3)	0.3263 (3)	0.0158 (8)	
C25	0.5112 (4)	0.2693 (4)	0.0096 (3)	0.0187 (8)	
H25A	0.4442	0.2121	0.0132	0.022*	
C26	0.5303 (5)	0.3467 (4)	−0.0766 (3)	0.0191 (8)	
H26A	0.4799	0.3399	−0.1311	0.023*	
C27	0.6228 (4)	0.4331 (3)	−0.0820 (3)	0.0178 (8)	
H27A	0.6357	0.4868	−0.1399	0.021*	
C28	0.6980 (4)	0.4406 (3)	−0.0005 (3)	0.0168 (8)	
C29	0.6754 (4)	0.3571 (3)	0.0821 (3)	0.0148 (8)	
C30	0.7935 (4)	0.5271 (3)	0.0033 (3)	0.0183 (8)	
H30A	0.8064	0.5861	−0.0512	0.022*	
C31	0.8670 (4)	0.5269 (3)	0.0841 (3)	0.0191 (8)	
H31A	0.9272	0.5870	0.0854	0.023*	
C32	0.8549 (4)	0.4373 (3)	0.1670 (3)	0.0163 (8)	
C33	0.7556 (4)	0.3548 (3)	0.1667 (3)	0.0146 (8)	
C34	0.9387 (4)	0.4249 (3)	0.2496 (3)	0.0180 (8)	
H34A	1.0080	0.4782	0.2540	0.022*	
C35	0.9176 (4)	0.3342 (4)	0.3233 (3)	0.0198 (8)	
H35A	0.9745	0.3235	0.3787	0.024*	
C36	0.8131 (4)	0.2570 (3)	0.3177 (3)	0.0180 (8)	
H36A	0.7990	0.1957	0.3702	0.022*	
Cl1	1.08237 (10)	0.70256 (8)	0.19629 (6)	0.0169 (2)	
Cl2	0.36235 (10)	0.30584 (8)	0.26606 (6)	0.0171 (2)	

### Atomic displacement parameters (Å^2^)

**Table:**

	*U*^11^	*U*^22^	*U*^33^	*U*^12^	*U*^13^	*U*^23^
Cu1	0.0201 (3)	0.0137 (3)	0.0081 (2)	−0.00360 (19)	−0.00410 (17)	0.00211 (18)
Cu2	0.0204 (3)	0.0143 (3)	0.0080 (2)	−0.00389 (19)	−0.00415 (17)	0.00174 (18)
O1	0.0206 (14)	0.0208 (15)	0.0089 (13)	−0.0035 (12)	−0.0059 (10)	0.0014 (11)
O2	0.0207 (14)	0.0191 (14)	0.0090 (13)	−0.0052 (11)	−0.0046 (10)	0.0005 (11)
O3	0.0220 (14)	0.0152 (14)	0.0125 (13)	−0.0042 (11)	−0.0042 (10)	0.0009 (11)
O4	0.0221 (14)	0.0176 (14)	0.0109 (13)	−0.0030 (11)	−0.0055 (10)	−0.0012 (11)
O5	0.0225 (14)	0.0175 (14)	0.0109 (13)	−0.0051 (11)	−0.0066 (10)	0.0004 (11)
O6	0.0242 (14)	0.0161 (14)	0.0127 (14)	−0.0039 (11)	−0.0060 (11)	0.0010 (11)
N1	0.0181 (16)	0.0155 (17)	0.0106 (15)	−0.0019 (13)	−0.0026 (12)	−0.0003 (13)
N2	0.0170 (16)	0.0169 (17)	0.0118 (16)	−0.0026 (13)	−0.0029 (12)	−0.0022 (13)
N3	0.0233 (17)	0.0152 (17)	0.0096 (15)	−0.0033 (13)	−0.0031 (12)	−0.0017 (13)
N4	0.0170 (16)	0.0146 (16)	0.0098 (15)	−0.0013 (13)	−0.0026 (12)	−0.0011 (13)
C1	0.0173 (18)	0.0158 (19)	0.0100 (18)	−0.0015 (15)	−0.0012 (14)	−0.0034 (15)
C2	0.0183 (19)	0.020 (2)	0.0125 (18)	−0.0014 (16)	−0.0040 (14)	−0.0023 (15)
C3	0.0199 (19)	0.018 (2)	0.0158 (19)	−0.0053 (16)	−0.0002 (15)	−0.0023 (16)
C4	0.0178 (19)	0.017 (2)	0.0120 (18)	−0.0015 (15)	−0.0031 (14)	−0.0006 (15)
C5	0.028 (2)	0.023 (2)	0.0125 (19)	−0.0068 (18)	−0.0092 (15)	0.0022 (16)
C6	0.0153 (18)	0.018 (2)	0.0099 (17)	0.0010 (15)	0.0005 (13)	−0.0034 (15)
C7	0.023 (2)	0.019 (2)	0.0118 (18)	−0.0029 (16)	−0.0053 (14)	0.0015 (15)
C8	0.025 (2)	0.020 (2)	0.0138 (19)	−0.0004 (17)	−0.0062 (15)	−0.0008 (16)
C9	0.023 (2)	0.020 (2)	0.0081 (18)	0.0024 (16)	−0.0032 (14)	0.0030 (15)
C10	0.0185 (19)	0.017 (2)	0.0111 (18)	0.0010 (15)	0.0014 (14)	−0.0032 (15)
C11	0.0178 (18)	0.0148 (19)	0.0103 (18)	0.0004 (15)	−0.0009 (14)	−0.0018 (15)
C12	0.022 (2)	0.0142 (19)	0.0152 (19)	−0.0010 (16)	−0.0018 (15)	0.0012 (15)
C13	0.0193 (19)	0.018 (2)	0.016 (2)	−0.0034 (16)	0.0007 (15)	−0.0026 (16)
C14	0.0193 (19)	0.016 (2)	0.0136 (19)	−0.0006 (15)	−0.0007 (14)	−0.0030 (15)
C15	0.0150 (17)	0.019 (2)	0.0087 (17)	−0.0016 (15)	0.0002 (13)	−0.0035 (15)
C16	0.0195 (19)	0.019 (2)	0.016 (2)	−0.0042 (16)	−0.0035 (15)	−0.0036 (16)
C17	0.024 (2)	0.027 (2)	0.0079 (18)	−0.0028 (17)	−0.0042 (15)	−0.0003 (16)
C18	0.0222 (19)	0.017 (2)	0.0083 (17)	−0.0006 (16)	−0.0052 (14)	0.0026 (15)
C19	0.0181 (18)	0.018 (2)	0.0070 (17)	−0.0006 (15)	−0.0020 (13)	−0.0022 (14)
C20	0.021 (2)	0.021 (2)	0.0139 (19)	−0.0013 (16)	−0.0058 (15)	−0.0037 (16)
C21	0.022 (2)	0.019 (2)	0.017 (2)	−0.0019 (16)	−0.0029 (15)	−0.0027 (16)
C22	0.0211 (19)	0.017 (2)	0.0119 (18)	−0.0032 (16)	0.0014 (14)	−0.0018 (15)
C23	0.029 (2)	0.020 (2)	0.0092 (18)	−0.0022 (17)	−0.0067 (15)	0.0029 (16)
C24	0.0172 (18)	0.0162 (19)	0.0132 (19)	0.0010 (15)	−0.0042 (14)	−0.0019 (15)
C25	0.023 (2)	0.020 (2)	0.0131 (19)	−0.0029 (16)	−0.0060 (15)	−0.0018 (16)
C26	0.024 (2)	0.023 (2)	0.0097 (18)	0.0001 (17)	−0.0049 (14)	−0.0024 (16)
C27	0.025 (2)	0.019 (2)	0.0068 (17)	0.0011 (16)	−0.0041 (14)	0.0031 (15)
C28	0.0168 (18)	0.019 (2)	0.0133 (19)	0.0022 (16)	−0.0023 (14)	−0.0015 (16)
C29	0.0167 (18)	0.0154 (19)	0.0118 (18)	0.0003 (15)	−0.0008 (14)	−0.0027 (15)
C30	0.0199 (19)	0.018 (2)	0.0141 (19)	−0.0019 (16)	0.0020 (15)	0.0036 (15)
C31	0.021 (2)	0.016 (2)	0.019 (2)	−0.0028 (16)	0.0012 (15)	−0.0013 (16)
C32	0.0207 (19)	0.0144 (19)	0.0124 (19)	−0.0006 (15)	0.0009 (14)	−0.0005 (15)
C33	0.0189 (19)	0.0146 (19)	0.0106 (18)	0.0000 (15)	−0.0048 (14)	−0.0033 (14)
C34	0.0209 (19)	0.017 (2)	0.016 (2)	−0.0015 (16)	−0.0053 (15)	−0.0031 (16)
C35	0.021 (2)	0.023 (2)	0.015 (2)	−0.0006 (17)	−0.0075 (15)	−0.0029 (16)
C36	0.025 (2)	0.015 (2)	0.0123 (18)	−0.0006 (16)	−0.0043 (15)	0.0012 (15)
Cl1	0.0197 (5)	0.0172 (5)	0.0129 (4)	−0.0019 (4)	−0.0013 (3)	−0.0008 (4)
Cl2	0.0205 (5)	0.0171 (5)	0.0127 (4)	−0.0019 (4)	−0.0027 (3)	−0.0008 (4)

### Geometric parameters (Å, °)

**Table:**

Cu1—N1	2.001 (3)	C10—C12	1.440 (5)
Cu1—N2	2.010 (3)	C11—C15	1.441 (5)
Cu1—O1	1.979 (3)	C12—C13	1.364 (5)
Cu1—O2	1.920 (3)	C12—H12A	0.9500
Cu1—Cl1	2.540 (1)	C13—C14	1.429 (5)
Cu2—O4	1.989 (3)	C13—H13A	0.9500
Cu2—O5	1.924 (3)	C14—C15	1.398 (5)
Cu2—N3	1.999 (3)	C14—C16	1.424 (5)
Cu2—N4	2.014 (3)	C16—C17	1.364 (5)
Cu2—Cl2	2.524 (1)	C16—H16A	0.9500
O1—C1	1.275 (5)	C17—C18	1.394 (6)
O2—C6	1.312 (5)	C17—H17A	0.9500
O3—C3	1.343 (5)	C18—H18A	0.9500
O3—C4	1.377 (4)	C19—C20	1.420 (5)
O4—C19	1.268 (5)	C19—C24	1.446 (5)
O5—C24	1.320 (5)	C20—C21	1.349 (6)
O6—C21	1.343 (5)	C20—H20A	0.9500
O6—C22	1.367 (5)	C21—H21A	0.9500
N1—C7	1.331 (5)	C22—C24	1.373 (5)
N1—C11	1.353 (5)	C22—C23	1.483 (5)
N2—C18	1.328 (5)	C23—H23A	0.9800
N2—C15	1.349 (5)	C23—H23B	0.9800
N3—C25	1.334 (5)	C23—H23C	0.9800
N3—C29	1.351 (5)	C25—C26	1.401 (5)
N4—C36	1.335 (5)	C25—H25A	0.9500
N4—C33	1.349 (5)	C26—C27	1.381 (6)
C1—C2	1.420 (5)	C26—H26A	0.9500
C1—C6	1.446 (5)	C27—C28	1.410 (5)
C2—C3	1.344 (5)	C27—H27A	0.9500
C2—H2A	0.9500	C28—C29	1.405 (5)
C3—H3A	0.9500	C28—C30	1.425 (5)
C4—C6	1.366 (5)	C29—C33	1.442 (5)
C4—C5	1.481 (5)	C30—C31	1.368 (6)
C5—H5A	0.9800	C30—H30A	0.9500
C5—H5B	0.9800	C31—C32	1.436 (5)
C5—H5C	0.9800	C31—H31A	0.9500
C7—C8	1.397 (5)	C32—C33	1.400 (5)
C7—H7A	0.9500	C32—C34	1.416 (5)
C8—C9	1.377 (6)	C34—C35	1.372 (6)
C8—H8A	0.9500	C34—H34A	0.9500
C9—C10	1.409 (5)	C35—C36	1.401 (5)
C9—H9A	0.9500	C35—H35A	0.9500
C10—C11	1.406 (5)	C36—H36A	0.9500
			
O2—Cu1—O1	85.57 (11)	C10—C12—H12A	119.6
O2—Cu1—N1	165.20 (13)	C12—C13—C14	121.3 (4)
O1—Cu1—N1	95.47 (12)	C12—C13—H13A	119.4
O2—Cu1—N2	93.73 (12)	C14—C13—H13A	119.4
O1—Cu1—N2	166.51 (13)	C15—C14—C16	115.9 (3)
N1—Cu1—N2	81.81 (13)	C15—C14—C13	119.3 (4)
O2—Cu1—Cl1	104.77 (9)	C16—C14—C13	124.7 (4)
O1—Cu1—Cl1	101.17 (9)	N2—C15—C14	124.8 (3)
N1—Cu1—Cl1	89.55 (10)	N2—C15—C11	115.8 (3)
N2—Cu1—Cl1	92.05 (10)	C14—C15—C11	119.5 (3)
O5—Cu2—O4	85.16 (11)	C17—C16—C14	118.8 (4)
O5—Cu2—N3	162.75 (13)	C17—C16—H16A	120.6
O4—Cu2—N3	94.60 (12)	C14—C16—H16A	120.6
O5—Cu2—N4	93.60 (12)	C16—C17—C18	120.9 (4)
O4—Cu2—N4	164.18 (13)	C16—C17—H17A	119.6
N3—Cu2—N4	81.93 (13)	C18—C17—H17A	119.6
O5—Cu2—Cl2	105.20 (9)	N2—C18—C17	121.8 (3)
O4—Cu2—Cl2	101.42 (9)	N2—C18—H18A	119.1
N3—Cu2—Cl2	91.78 (10)	C17—C18—H18A	119.1
N4—Cu2—Cl2	94.13 (10)	O4—C19—C20	125.1 (3)
C1—O1—Cu1	109.2 (2)	O4—C19—C24	117.4 (3)
C6—O2—Cu1	109.6 (2)	C20—C19—C24	117.5 (3)
C3—O3—C4	119.9 (3)	C21—C20—C19	119.2 (4)
C19—O4—Cu2	109.7 (2)	C21—C20—H20A	120.4
C24—O5—Cu2	109.4 (2)	C19—C20—H20A	120.4
C21—O6—C22	120.5 (3)	O6—C21—C20	123.0 (4)
C7—N1—C11	118.5 (3)	O6—C21—H21A	118.5
C7—N1—Cu1	128.6 (3)	C20—C21—H21A	118.5
C11—N1—Cu1	112.4 (2)	O6—C22—C24	120.5 (3)
C18—N2—C15	117.8 (3)	O6—C22—C23	113.1 (3)
C18—N2—Cu1	129.3 (3)	C24—C22—C23	126.3 (4)
C15—N2—Cu1	112.4 (2)	C22—C23—H23A	109.5
C25—N3—C29	118.6 (3)	C22—C23—H23B	109.5
C25—N3—Cu2	128.5 (3)	H23A—C23—H23B	109.5
C29—N3—Cu2	112.7 (2)	C22—C23—H23C	109.5
C36—N4—C33	118.2 (3)	H23A—C23—H23C	109.5
C36—N4—Cu2	129.4 (3)	H23B—C23—H23C	109.5
C33—N4—Cu2	112.4 (2)	O5—C24—C22	122.9 (3)
O1—C1—C2	124.9 (3)	O5—C24—C19	117.9 (3)
O1—C1—C6	117.7 (3)	C22—C24—C19	119.2 (3)
C2—C1—C6	117.4 (3)	N3—C25—C26	122.1 (4)
C3—C2—C1	119.4 (4)	N3—C25—H25A	119.0
C3—C2—H2A	120.3	C26—C25—H25A	119.0
C1—C2—H2A	120.3	C27—C26—C25	119.6 (4)
O3—C3—C2	123.1 (3)	C27—C26—H26A	120.2
O3—C3—H3A	118.4	C25—C26—H26A	120.2
C2—C3—H3A	118.4	C26—C27—C28	119.2 (3)
C6—C4—O3	120.9 (3)	C26—C27—H27A	120.4
C6—C4—C5	126.1 (4)	C28—C27—H27A	120.4
O3—C4—C5	113.0 (3)	C29—C28—C27	117.2 (4)
C4—C5—H5A	109.5	C29—C28—C30	118.5 (4)
C4—C5—H5B	109.5	C27—C28—C30	124.4 (3)
H5A—C5—H5B	109.5	N3—C29—C28	123.3 (4)
C4—C5—H5C	109.5	N3—C29—C33	116.2 (3)
H5A—C5—H5C	109.5	C28—C29—C33	120.5 (3)
H5B—C5—H5C	109.5	C31—C30—C28	121.2 (3)
O2—C6—C4	123.1 (3)	C31—C30—H30A	119.4
O2—C6—C1	117.7 (3)	C28—C30—H30A	119.4
C4—C6—C1	119.2 (3)	C30—C31—C32	121.3 (4)
N1—C7—C8	122.3 (4)	C30—C31—H31A	119.4
N1—C7—H7A	118.8	C32—C31—H31A	119.4
C8—C7—H7A	118.8	C33—C32—C34	116.9 (3)
C9—C8—C7	119.6 (4)	C33—C32—C31	118.6 (4)
C9—C8—H8A	120.2	C34—C32—C31	124.5 (4)
C7—C8—H8A	120.2	N4—C33—C32	124.1 (3)
C8—C9—C10	119.3 (4)	N4—C33—C29	116.0 (3)
C8—C9—H9A	120.4	C32—C33—C29	119.8 (3)
C10—C9—H9A	120.4	C35—C34—C32	118.4 (4)
C11—C10—C9	117.1 (3)	C35—C34—H34A	120.8
C11—C10—C12	118.4 (3)	C32—C34—H34A	120.8
C9—C10—C12	124.5 (4)	C34—C35—C36	120.8 (4)
N1—C11—C10	123.1 (3)	C34—C35—H35A	119.6
N1—C11—C15	116.2 (3)	C36—C35—H35A	119.6
C10—C11—C15	120.6 (3)	N4—C36—C35	121.5 (3)
C13—C12—C10	120.9 (3)	N4—C36—H36A	119.2
C13—C12—H12A	119.6	C35—C36—H36A	119.2
			
O2—Cu1—O1—C1	−3.4 (3)	C12—C10—C11—C15	0.0 (6)
N1—Cu1—O1—C1	−168.6 (3)	C11—C10—C12—C13	−0.1 (6)
N2—Cu1—O1—C1	−91.0 (6)	C9—C10—C12—C13	−179.7 (4)
Cl1—Cu1—O1—C1	100.8 (2)	C10—C12—C13—C14	−1.7 (6)
O1—Cu1—O2—C6	4.3 (3)	C12—C13—C14—C15	3.4 (6)
N1—Cu1—O2—C6	99.0 (5)	C12—C13—C14—C16	−177.6 (4)
N2—Cu1—O2—C6	170.8 (3)	C18—N2—C15—C14	0.8 (6)
Cl1—Cu1—O2—C6	−96.1 (2)	Cu1—N2—C15—C14	−171.9 (3)
O5—Cu2—O4—C19	4.9 (3)	C18—N2—C15—C11	−177.9 (4)
N3—Cu2—O4—C19	167.6 (3)	Cu1—N2—C15—C11	9.4 (4)
N4—Cu2—O4—C19	91.1 (5)	C16—C14—C15—N2	−1.2 (6)
Cl2—Cu2—O4—C19	−99.6 (2)	C13—C14—C15—N2	177.9 (4)
O4—Cu2—O5—C24	−5.7 (2)	C16—C14—C15—C11	177.5 (3)
N3—Cu2—O5—C24	−95.7 (4)	C13—C14—C15—C11	−3.4 (6)
N4—Cu2—O5—C24	−169.9 (3)	N1—C11—C15—N2	−1.3 (5)
Cl2—Cu2—O5—C24	94.8 (2)	C10—C11—C15—N2	−179.5 (3)
O2—Cu1—N1—C7	−105.4 (5)	N1—C11—C15—C14	179.9 (3)
O1—Cu1—N1—C7	−12.0 (4)	C10—C11—C15—C14	1.7 (6)
N2—Cu1—N1—C7	−178.7 (4)	C15—C14—C16—C17	0.3 (6)
Cl1—Cu1—N1—C7	89.2 (3)	C13—C14—C16—C17	−178.8 (4)
O2—Cu1—N1—C11	83.0 (5)	C14—C16—C17—C18	0.8 (6)
O1—Cu1—N1—C11	176.4 (3)	C15—N2—C18—C17	0.4 (6)
N2—Cu1—N1—C11	9.7 (3)	Cu1—N2—C18—C17	171.7 (3)
Cl1—Cu1—N1—C11	−82.5 (3)	C16—C17—C18—N2	−1.3 (6)
O2—Cu1—N2—C18	12.1 (4)	Cu2—O4—C19—C20	176.2 (3)
O1—Cu1—N2—C18	98.7 (6)	Cu2—O4—C19—C24	−3.1 (4)
N1—Cu1—N2—C18	177.9 (4)	O4—C19—C20—C21	−177.4 (4)
Cl1—Cu1—N2—C18	−92.9 (3)	C24—C19—C20—C21	1.8 (6)
O2—Cu1—N2—C15	−176.2 (3)	C22—O6—C21—C20	0.3 (6)
O1—Cu1—N2—C15	−89.6 (6)	C19—C20—C21—O6	−1.4 (6)
N1—Cu1—N2—C15	−10.4 (3)	C21—O6—C22—C24	0.2 (6)
Cl1—Cu1—N2—C15	78.8 (3)	C21—O6—C22—C23	179.6 (3)
O5—Cu2—N3—C25	100.3 (5)	Cu2—O5—C24—C22	−174.0 (3)
O4—Cu2—N3—C25	11.8 (4)	Cu2—O5—C24—C19	5.8 (4)
N4—Cu2—N3—C25	176.3 (4)	O6—C22—C24—O5	−179.9 (3)
Cl2—Cu2—N3—C25	−89.8 (4)	C23—C22—C24—O5	0.9 (6)
O5—Cu2—N3—C29	−82.9 (5)	O6—C22—C24—C19	0.3 (6)
O4—Cu2—N3—C29	−171.4 (3)	C23—C22—C24—C19	−178.9 (4)
N4—Cu2—N3—C29	−7.0 (3)	O4—C19—C24—O5	−1.8 (5)
Cl2—Cu2—N3—C29	87.0 (3)	C20—C19—C24—O5	178.9 (3)
O5—Cu2—N4—C36	−11.7 (4)	O4—C19—C24—C22	178.0 (4)
O4—Cu2—N4—C36	−96.7 (5)	C20—C19—C24—C22	−1.3 (6)
N3—Cu2—N4—C36	−174.9 (4)	C29—N3—C25—C26	2.1 (6)
Cl2—Cu2—N4—C36	93.9 (3)	Cu2—N3—C25—C26	178.7 (3)
O5—Cu2—N4—C33	171.0 (3)	N3—C25—C26—C27	−2.5 (6)
O4—Cu2—N4—C33	86.0 (5)	C25—C26—C27—C28	0.8 (6)
N3—Cu2—N4—C33	7.8 (3)	C26—C27—C28—C29	1.0 (6)
Cl2—Cu2—N4—C33	−83.5 (3)	C26—C27—C28—C30	−178.5 (4)
Cu1—O1—C1—C2	−177.5 (3)	C25—N3—C29—C28	−0.1 (6)
Cu1—O1—C1—C6	1.9 (4)	Cu2—N3—C29—C28	−177.2 (3)
O1—C1—C2—C3	177.2 (4)	C25—N3—C29—C33	−177.8 (3)
C6—C1—C2—C3	−2.2 (6)	Cu2—N3—C29—C33	5.1 (4)
C4—O3—C3—C2	0.2 (6)	C27—C28—C29—N3	−1.5 (6)
C1—C2—C3—O3	1.8 (6)	C30—C28—C29—N3	178.1 (4)
C3—O3—C4—C6	−1.8 (6)	C27—C28—C29—C33	176.1 (4)
C3—O3—C4—C5	178.3 (3)	C30—C28—C29—C33	−4.3 (6)
Cu1—O2—C6—C4	176.1 (3)	C29—C28—C30—C31	2.7 (6)
Cu1—O2—C6—C1	−4.5 (4)	C27—C28—C30—C31	−177.7 (4)
O3—C4—C6—O2	−179.3 (3)	C28—C30—C31—C32	2.2 (6)
C5—C4—C6—O2	0.6 (7)	C30—C31—C32—C33	−5.5 (6)
O3—C4—C6—C1	1.3 (6)	C30—C31—C32—C34	173.4 (4)
C5—C4—C6—C1	−178.8 (4)	C36—N4—C33—C32	−2.0 (6)
O1—C1—C6—O2	1.8 (5)	Cu2—N4—C33—C32	175.7 (3)
C2—C1—C6—O2	−178.8 (3)	C36—N4—C33—C29	175.1 (3)
O1—C1—C6—C4	−178.8 (4)	Cu2—N4—C33—C29	−7.3 (4)
C2—C1—C6—C4	0.6 (6)	C34—C32—C33—N4	1.8 (6)
C11—N1—C7—C8	0.9 (6)	C31—C32—C33—N4	−179.2 (4)
Cu1—N1—C7—C8	−170.3 (3)	C34—C32—C33—C29	−175.1 (3)
N1—C7—C8—C9	0.3 (6)	C31—C32—C33—C29	3.9 (6)
C7—C8—C9—C10	−0.6 (6)	N3—C29—C33—N4	1.5 (5)
C8—C9—C10—C11	−0.3 (6)	C28—C29—C33—N4	−176.2 (4)
C8—C9—C10—C12	179.3 (4)	N3—C29—C33—C32	178.7 (4)
C7—N1—C11—C10	−1.9 (6)	C28—C29—C33—C32	1.0 (6)
Cu1—N1—C11—C10	170.6 (3)	C33—C32—C34—C35	−0.1 (6)
C7—N1—C11—C15	179.9 (3)	C31—C32—C34—C35	−179.0 (4)
Cu1—N1—C11—C15	−7.5 (4)	C32—C34—C35—C36	−1.4 (6)
C9—C10—C11—N1	1.6 (6)	C33—N4—C36—C35	0.4 (6)
C12—C10—C11—N1	−178.0 (4)	Cu2—N4—C36—C35	−176.8 (3)
C9—C10—C11—C15	179.7 (4)	C34—C35—C36—N4	1.3 (6)
